# Central Composite Design for Optimizing the Biosynthesis of Silver Nanoparticles using *Plantago major* Extract and Investigating Antibacterial, Antifungal and Antioxidant Activity

**DOI:** 10.1038/s41598-020-66357-3

**Published:** 2020-06-15

**Authors:** Ghazal Nikaeen, Saeed Yousefinejad, Samane Rahmdel, Fayezeh Samari, Saeideh Mahdavinia

**Affiliations:** 1 Research Center for Health Sciences, Institute of Health, Department of Occupational Health Engineering, School of Health, Shiraz University of Medical Sciences, Shiraz, Iran; 2 Nutrition Research Center, School of Nutrition and Food Sciences, Shiraz University of Medical Sciences, Shiraz, Iran; 3 Department of Chemistry, Faculty of Sciences, University of Hormozgan, 71961 Bandar Abbas, Iran

**Keywords:** Chemistry, Nanoscience and technology

## Abstract

Central composite design (CCD) was applied to optimize the synthesis condition of silver nanoparticles (AgNPs) using the extract of *Plantago major* (*P. major*) seeds via a low cost and single-step process. The aqueous seed extract was applied as both reducing element and capping reagent for green production of AgNPs. Five empirical factors of synthesis including temperature (*Temp*), pH, volume of *P. major* extract (*V*_*ex*_), volume of AgNO_3_ solution (*V*_*Ag*_) and synthesis time were used as independent variables of model and peak intensity of Surface Plasmon Resonance (SPR) originated from NPs as the dependent variable. The predicted optimal conditions was determined to be: *Temp* = 55 °C, *pH* = 9.9,*V*_*ex*_ = 1.5 mL, *V*_*Ag*_ = 30 mL, *time* = 60 min. The characterization of the prepared AgNPs at these optimum conditions was conducted by Fourier transform infrared spectroscopy (FTIR), dynamic light scattering (DLS), transmission electron microscopy (TEM) and X-ray diffraction (XRD) to determine the surface bio-functionalities. Bio-activity of these AgNPs against bacteria and fungi were evaluated based on its assay against *Micrococcus luteus, Escherichia coli* and *Penicillium digitatum*. Furthermore, antioxidant capacity of these NPs was checked using the ferric reducing antioxidant power (FRAP) assay.

## Introduction

Nanotechnology is an important field of modern research which has been the principal of various technologies and main innovations; and is expected to be the basis of many other outstanding innovations in future. Because of importance and applications, research and development in nanoscience has been grown throughout the world during a short period of time^1–5^. One of the major outputs of these studies is the introduction of new kind of materials in the nano-scale, including nonofilms, nanoparticles, nanowires and nanoclusters. Among different kinds of nanomaterials, metal nanoparticles are well-known due to their interesting properties in optical, electrical, magnetic and catalytic aspects^6^.

Metal nanomaterials prepared from the noble metals, in particular gold or silver, have attracted great interests due to their generated strong Surface Plasmon Resonance (SPR)^7^. This fact should be noted that the SPR is a sensitive indicator of nanostructures geometry, which can be applied to follow the systematic and controlled variations in their geometry during preparation. SPR has been also widely used in sensing field which is known as “plasmonics”^8^.

During the past few decades, the exceptional properties of AgNPs have introduced them as reliable candidate for using in different fields such as biomedical^9^, antimicrobial and anti-infection medical device^10,11^, water treatment^12^, drug delivery^13^, agricultural applications^14^, surface-enhanced Raman scattering (SERS)^15^ and electromagnetic interference shielding^16^.

A number of approaches has been proposed to prepare AgNPs such as, chemical reduction of silver salts in solution^17^, photochemical and chemical process in reverse micelles^18^, radiation assisted approaches^19,20^, thermal decomposition of silver polymers and compounds^21,22^, electrochemical synthesis^23^, sonochemical processes^24^ and microwave assisted approaches^25^.

Simpler methods based on green chemistry routes has been suggested for the preparation of AgNPs such as using plant leaf extract^26–28^, algae^29^, bacteria^30^ and fungi^31^ which can be done with low-cost and energy-efficient strategies and without accumulating any enormous amount of redundant compounds and toxic chemicals in the environment^11^. Among various green approaches for the preparation of AgNPs, using plant extracts is more abundant which actually apply as reducing agents of Ag^+^ and stabilizing molecules for the produced Ag^0^ NPs. Suggesting of potent plants and herbal extracts for the preparation of AgNPs can help industries and researchers to make AgNPs in large production scales. For this goal, synthesis of AgNPs using *Plantago major* (*P. major*) seeds was performed in this research.

*P. major*, usually named *great plantain*, is known as a medicinal plant with widespread applications and is categorized in the Plantaginaceae family^32^. *P. major* is also a common plant in Traditional Persian Medicine and is known as “Barhang” or “Lesan-ol-haml”^33^. It is noted in literature that *P. major* has grown since 4000 years ago in different areas specially in Europe, Asia and America^34^. In addition, to wide access to *P. major*, it has been applied at ancient times in various points of Iran^34^. *P. major* obtains a large amount of seeds which have been utilized for a long time as an immune-modulating, anti-infective, analgesic, anti-inflammatory, anti-ulcerogenic, anti-microbial, anti-cancer and antioxidant agent^35^. The *P. major* seed has been applied also for wound curing purposes. There are a few reports on the application of *P. major* in the synthesis of AgNPs such as the research conducted by Romeh *et al*. in which AgNPs was prepared using *P. major* leaves and has been applied on the fipronil degradation in water media^36^. The other study was performed by SobhaniPoor *et al*. on the application of seed extract of *P. major* in green synthesis of AgNPs and investigation of its cytotoxic effect on MCF-7 cancer cells^37^. However, no focus was done on the effects of different factors or their interactions during the biosynthesis of AgNPs in these studies.

In the present research, a low cost and simple process and environmentally friendly method for preparation of AgNPs was suggest using the seeds extract of *P. major*. Here also, the influence of different experimental variables, such as AgNO_3_ concentration, amount of *P. major* seeds extract, pH, temperature and synthesis time were studied using response surface methodology (RSM). It should be noted that application of RSM for the experimental design of the synthesis process can be important and useful for the exact and fast optimization of conditions and preparation of these NPs in high scale by decreasing trial-and-error runs^38,39^. Report on the application of experimental design in green synthesis of AgNPs is rare^40,41^ and this is a comprehensive investigation on different experimental parameters in the biosynthesis of AgNPs based on herbal extracts using central composite design (CCD). The as-synthesized AgNPs were characterized with different spectroscopic and microscopic methods and then were evaluated for antibacterial, antifungal and antioxidant applications.

## Materials and methods

### Chemicals and Plants

*P. major* seed samples were collected from Shiraz (Fars province, south of Iran). All the utilized chemicals were of analytical grade and no further purification were done before application in experiments. The utilized water in all steps of synthesis and activity evaluations was deionized distilled water prepared by a Millipore system. Silver nitrate (AgNO_3_), methylene blue (MB), rhodamine B (RhB) and sodium borohydride (NaBH_4_) were prepared from Merck Company (Germany).

Aqua regia was used to wash all glassware and then was rinsed several times with distilled water and finally with deionized water. HNO_3_ (0.1 M) and NaOH (0.1 M) solutions were used for the pH adjustments.

### Instruments

The UV–Vis absorbance measurements were done using a Carry 60 UV–vis spectrophotometer (Agilent, USA) by a quartz cuvette (10 mm). The X-ray diffraction (XRD) were performed by the XRD Bruker D8 Advance powder diffractometer (Bruker, Germany) by applying the reflection mode with Cu-K_α_ radiation (λ = 1.5406 Å). The Fourier Transform-Infra Red (FT-IR) spectra were collected at room temperature using a “Spectrum RXI” Perkin-Elmer FT-IR spectrophotometer (PerkinElmer, USA) after tableting the AgNPs powder. The distribution of size and volume of NPs were also determined using Dynamic Light Scattering (DLS) using HORIBA SZ-100 nanoparticle analyzer (HORIBA, Japan).

The morphology of the particles was investigated using the Transmission Electron Microscopic examination (TEM). For this study, colloidal AgNPs were sonicated and a thin film was formed on the carbon coated grid Cu Mesh 300. TEM measurements were done using a Zeiss–EM10C microscope (Zeiss, Germany) operated at an accelerating voltage of 80 kV. The pH adjustment during synthesis was performed using a Metrohm 827 pH-meter equipped with a combined glass electrode (Metrohm, Switzer-land).

### **Preparation of*Plantago major*extract**

The seeds of *P. major* were prepared from local market (Shiraz, Iran) and were fast-washed with distilled water to eliminate the dusts and impurities. The clean and dried seeds were powdered mechanically. 20.0 g powder of *P. major* seeds was mixed in 100 mL of deionized water in 500 mL flask; and heated at 80 °C for 30 min by using a heater-magnetic stirrer. After cooling, the resulted solution was initially centrifuged and filtered through normal textile filter to eliminate the big particles; then the obtained extract was filtered with filtration paper (Whatman, No. #1). The filtered *P. major* extract was stored in the refrigerator at 4 °C before use. The extract solution was not utilized more than one week. It is clear that the *P. major* extract was applied as the stabilizing and reducing agent during the production of silver NPs.

### **Preparation of AgNPs using*Plantago major*extract**

In the current research, the solution of silver nitrate salt in deionized water was applied as the metal source (Ag). The reaction vessel contained the determined volume (V_e_, mL) of the extract in desired volume of AgNO_3_ solution 1 mM (V_Ag_, ml) under stirring at reaction temperature (Temp, °C) and desired reaction time (t, min) for the reduction of Ag^+^ to Ag°. The reaction was continued until the color of the solution became reddish-brown that showed the construction of AgNPs. The effect of pH on the synthesis of AgNPs was first studied by fixing the pH of the reaction media to 3.0, 5.0, 7.0, 9.0, 10 and 11.0 to have an estimate regarding the useful range of pH. Then the effect of temperature, pH, extract content, concentration of AgNO_3_ salt and mixing time were evaluated simultaneously using response surface methodology with CCD. The exact amounts of reagents and compounds, finally utilized for synthesis of AgNPs, were obtained during optimization with CCD and are reported and explained in the “Results and Discussion” section but is briefly denoted hare: *Temp* = 55 °C, *pH* = 9.9,*V*_*ex*_ = 1.5 mL, *V*_*Ag*_ = 30 mL, *time* = 60 min.

### Optimization of synthesis conditions using CCD

As it was noted previously, in order to obtain optimum synthesis conditions for preparation of AgNPs, a CCD has been applied. This method has been used to achieve the best possible SPR intensity as a confirmation of NPs production. For all UV-Vis spectrophotometric studies, equivalent volume of the suspension (0.5 ml) were diluted with a constant volume (=2 ml) of deionized water and subsequently all measurements were done at room temperature. The design was done based on five levels for each noted factor in single-block mode and the obtained 32 runs was performed in a random order to minimize the effect of uncontrolled variables on the quality of NPs (followed by SPR).

### Antibacterial activity

The antimicrobial activity of AgNPs against Gram-positive (*Micrococcus luteus* ATCC 9341) and Gram-negative (*Escherichia coli* ATCC 25922) indicator bacteria was first evaluated using the disk diffusion method described by Kumar *et al*.^42^. In brief, the overnight culture of bacterial strains was adjusted to a 0.5 McFarland turbidity standard (1.5 × 10^8^ cfu/mL). The inoculum was then added to the molten brain heart infusion (BHI) agar (Merck, Darmstadt, Germany) to achieve a final concentration of about 10^6^ cfu/mL. Afterwards, 6 mm-diameter blank disks were placed onto the seeded agar plates and 10 µL aliquots of different concentrations of AgNPs (full strength, 50%, 25%, 12.5%, 6.25%, and 3.125% v/v) as well as *P. major* extract were added to each disk. After 24 h incubation at 37 °C, the growth inhibition zone diameters were measured. Standard gentamicin disks (10 µg) were used as positive control. All tests were conducted in triplicate.

The minimal inhibitory concentrations (MIC) of AgNPs were also determined for test organisms using a broth microdilution method. The full-strength concentration of AgNPs (10 mM or 1078 µg/mL; which is shown as 100% in this research) was two-fold serially diluted in the wells of a microtiter plate containing 70 µL of BHI broth to achieve reduced concentrations (50%, 25%, 12.5%, 6.25%, and 3.125% v/v). Following addition of resazurin solution (final concentration of 1 mg/mL) and bacterial suspensions (final concentration of 10^6^ cfu/mL) to each well, the plate was incubated at 37 °C for 24 h. A set of controls were used in this experiment: a) the culture medium; b) the culture medium inoculated with test strains; c) the culture medium mixed with *P. major* extract and inoculated with test strains; and d) the culture medium mixed with AgNPs or *P. major* extract without bacteria. Bacterial growth was verified by color change from purple-blue to pink or colorless. The lowest concentration without any discoloration was considered as MIC value^43^. The experiments were carried out in 2 replicates.

### Antifungal activity

A disc diffusion assay was also applied to evaluate the antifungal activity of biosynthesized AgNPs against *Penicillium digitatum* IR1037c (*P*. *digitatum*). The conidia suspension was prepared by washing the surface of potato dextrose agar (PDA) containing a 7-day old culture of *P*. *digitatum* with 2 mL of normal saline (9 g/L NaCl). The suspension was then adjusted to 10^6^ conidia/mL using a Neubauer’s chamber and seeded in PDA to achieve a final concentration of 10^4^ conidia/mL. Blank disks were placed on the agar surface and after adding AgNPs or *P. major* extract (10 µL), the plates were incubated at 25 °C for 48 h^44^.

### Ferric reducing antioxidant assay for antioxidant activity

The antioxidant potential of *Plantago major* extract and AgNPs were checked using an *in vitro* assay, using ferric-reducing antioxidant power (FRAP)^45^. In FRAP test, the principal is on the potential of antioxidant compounds for reduction of the intense blue ferric tripyridyltriazine complex and formation of its ferrous complex as the product, which can lead to change the absorbance of ferric complex at 593 nm. The preparation of the required reagent for FRAP was done daily by adding acetate buffer (pH 3.6, 300 mM) to 10 mM solution of tripyridyl-s-triazine (TPTZ) in 40 mM HCl and 20 mM FeCl_3_.6H_2_O. The ratio of TPTZ: HCl: FeCl_3_.6H_2_O was 10:1:1 and was kept at 37 °C. Stock concentration of each target sample (*P. major* extract and prepared AgNPs) was firstly prepared in deionized water (1078 μg/mL) and different concentrations of NPs or *P. major* extract were prepared by dilution (808.5, 539, 269.5, 134.75 μg/mL). Then, 1.5 ml of the FRAP reagent was mixed with 50 μl of target sample and the mixture was then retained at 37 °C for 10 min in the dark. Here, excess amount of Fe(III) was utilized, and the color formation due to the construction of Fe(II)-TPTZ was the rate-limiting criteria to show the reducing power of the extract or investigated AgNPs. In the FRAP assay for each target sample, the blank solution was prepared with just FRAP reagent. In all runs of assay, the final absorbance of sample (i.e.: FRAP reagent + target solution) was corrected by subtraction of the blank absorbance. The calibration or standard curve of this assay was prepared using a set of solution with known concentrations of FeSO_4_ (100 to 2000 µM). All the obtained values in this assay were reported as mM of ferrous ion (Fe (II)) equivalent.

## Results and discussion

### Response surface methodology

In this work, CCD strategy was used to find optimum experimental condition for green synthesis of AgNPs. CCD has been employed to achieve the best SPR band of synthesized AgNPs with considering of the peak intensity. The goal was the investigation of the five empirical factors of synthesis including temperature (*Temp*), *pH*, volume of extract used for synthesis (*V*_*ex*_), volume of 1 mM AgNO_3_ solution (*V*_*Ag*_) and synthesis time (*time*).

**Figure 1:**
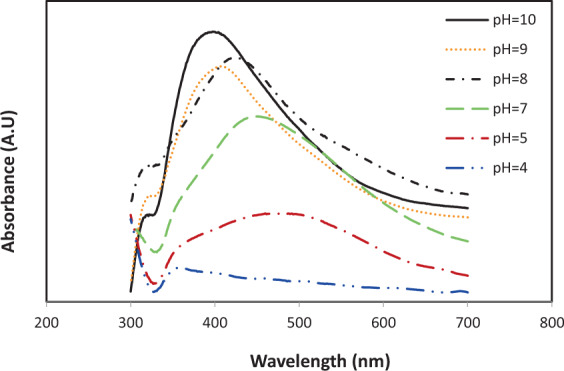
Effect of pH on the Plasmon resonance spectra of AgNPs.

It is worthy to note that based on our previous studies, pH is one of the most effective variables in synthesis of AgNPs. Thus, to determine the operational value range of pH, a serie of synthesis was done by one-at-a-time approach with varying the pH and constant amounts of other factors (*Temp* = 40 °C, *V*_*ex*_ = 2 ml, *V*_*Ag* (0.1mM)_=25 ml and time=60 min). As it is represented in Fig. 1, by increasing pH the quality of synthesis was enhanced and acidic pH (3.0 and 4.0) had no good results and doesn’t show SPR which can be related to the suppression of the AgNPs construction. At pH 5.0, as an acidic media, a broad SPR peak was observed with λ_max_ at 490 nm which can be due to increase in NPs size because of their agglomeration in acidic pH^46^. As it is observed in Fig. 1 in alkaline and neutral pH, the construction of particles was instantaneous which led to higher intensity in SPR band. This can be related to the ionization of the phenolic functional groups in the utilized seed extract and enhancement its reduction ability^47^. By increasing the pH from 7.0 to alkaline values, the target SPR was become narrower with blue shift (to lower wavelengths).

Thus the pH in neutral to alkaline range (7, 8.5 and 10) was the main levels which were used in CCD. Also two syntheses was done in lower and higher pH as their boundaries points (−α and +α), i.e in 5.5 and 11.5 respectively. Five experimental factors used for biosynthesis of AgNPs and their corresponding five levels (3 levels and 2 boundary levels) are represented in Table 1.

**Table 1:** Experimental factors for biosynthesis of AgNPs and corresponding levels.

Independent variable	Variable levels				
	Star-low (−α) (−2)	Low (−1)	Center (0)	High (+1)	Star-high (+α) (+2)
Temperature (°C)	10	25	40	55	70
pH	5.5	7	8.5	10	11.5
V_ex_ (ml)	0.5	1.5	2.5	3.5	4.5
V_ex_ (ml)	15	20	25	30	35
Time (min)	0	60	120	180	240

Table 2 represents 32 runs which has been designed by CCD and was made of a 2^4^ full factorial design. 16 experiments of these 32 were the factorial runs, which were included as the cubic points, and 6 replication runs were suggested in center points of levels, which were used for clarification of pure error. In addition, 8 axial runs were included as star points of the suggested CCD. To have a rotatable design for experiments, the α star points have been adjusted in a value of 2.0.

**Table 2:** Details of design runs for synthesis of AgNPs with the corresponding responses (absorbance intensity of the SPR).

Run	Temperature (°C)	pH	Extract Volume (ml)	Ag salt volume (ml)	Time (min)	Absorbance Intensity
**1**	40	8.5	2.5	25	120	0.840
**2**	40	8.5	2.5	25	120	0.653
**3**	40	8.5	2.5	25	120	0.763
**4**	55	10	3.5	30	180	1.187
**5**	25	7	3.5	20	60	0.070
**6**	55	7	3.5	30	60	1.058
**7**	40	8.5	2.5	25	120	1.131
**8**	25	10	3.5	30	60	0.970
**9**	25	10	1.5	30	180	1.173
**10**	55	7	3.5	20	180	0.772
**11**	40	8.5	0.5	25	120	1.517
**12**	70	8.5	2.5	25	120	1.460
**13**	40	8.5	4.5	25	120	1.181
**14**	25	7	1.5	20	180	0.846
**15**	40	5.5	2.5	25	120	0.521
**16**	40	8.5	2.5	35	120	0.958
**17**	25	7	3.5	30	180	0.855
**18**	25	10	3.5	20	180	1.341
**19**	55	7	1.5	20	60	0.916
**20**	55	10	1.5	30	60	1.627
**21**	55	7	1.5	30	180	1.130
**22**	40	8.5	2.5	25	120	1.106
**23**	40	8.5	2.5	25	120	0.970
**24**	40	8.5	2.5	25	240	0.867
**25**	55	10	1.5	20	180	1.083
**26**	55	10	3.5	20	60	1.162
**27**	40	8.5	2.5	15	120	0.917
**28**	40	11.5	2.5	25	120	0.870
**29**	40	8.5	2.5	25	0	0.742
**30**	25	10	1.5	20	60	1.108
**31**	25	7	1.5	30	60	1.366
**32**	10	8.5	2.5	25	120	0.741

“Peak intensity” of the SPR was the utilized as responses of the CCD design and modeling which resulted from recording the absorbance of 32 synthesized beaches of AgNPs at 490 nm.

### Statistical analysis of CCD

To start the statistical analysis using a multivariate optimization approach the first step was construction of a regression model between the SPR intensity (target response) and the experimental factors as the independent variables of model. For this goal, various linear and polynomial regression models were evaluated and different statistical parameters such as Fisher F-value using F-test (for both regression and lack of fit), squared correlation coefficient (*R*^2^), adjusted R^2^ (*R*^2^_*adj*_) and the R^2^ of prediction (*R*^2^_*pred*_) were utilized to select the best regression model. Finally based on these statistics, a quadratic model was proposed to correlate the relationship between the experimental factors and the response (peak intensity of SPR). Since the goal of this part was finding conditions for obtaining maximum SPR intensity, the analysis of variance (ANOVA) was constructed for this goal which is represented in more details in Table 3. According to Table 3, the performance parameters obtained for the quadratic full model (FM) including all experimental variables (*Temp*, *V*_*ex*_, *V*_*Ag*_, and *time*), their binary interactions (*Temp*. × *pH*, *Temp*. × *V*_*ex*_, *Temp*. × *V*_*Ag*_, *Temp*. ×*time*, *pH* × *V*_*ex*_, *pH* × *V*_*Ag*_, *pH* × *time*, *V*_*ex*_ *× V*_*Ag*_*, V*_*ex*_ *× time, V*_*Ag*_ *× time*) and self-interactions (*Temp*^2^*, V*_*ex*_^2^*, V*_*Ag*_^2^, and *time*^2^). The obtained model in terms of coded levels (−2, −1, 0, +1, +2) is shown in Eq. (1):

1 $$\begin{array}{rcl}{\rm{SPR}}\,{\rm{Intensity}} & = & +0.90+0.11\,Temp+0.14pH\,\mbox{--}\,0.10{V}_{ex}+0.090{V}_{Ag}+0.015\,time\\  &  & \,\mbox{--}\,0.017\,Temp\times pH+0.042\,Temp\times {V}_{ex}+0.004231\,Temp\times {V}_{Ag}\,\mbox{--}\,0.081\,Temp\,\times \,time\\  &  & +0.073pH\times {V}_{ex}\,\mbox{--}\,0.096\,pH\,\times \,{V}_{Ag}\,\mbox{--}\,0.017\,pH\,\times \,time\mbox{--}0.039\,{V}_{ex}\times {V}_{Ag}+0.10\,{V}_{ex}\,\times \,time\\  &  & \,\mbox{--}\,0.091{V}_{Ag}\times \,time+0.056\,Tem{p}^{2}\,\mbox{--}\,0.045\,p{H}^{2}+0.12\,{V}_{ex}2+0.015\,{V}_{Ag}2\,\mbox{--}\,0.018\,tim{e}^{2}\end{array}$$

**Table 3:** ANOVA and model performance of full quadratic model and the refined model after variable selection.

Analysis of variance (ANOVA)										
Source of variation	Sum of square		DF		Mean square		F		p-value	
	FM	MM	FM	RM	FM	RM	FM	RM	FM	RM
A (*Temp*)	0.29	0.29	1	1	0.29	0.29	8.74	10.92	0.0131	0.0037
B (*pH*)	0.46	0.46	1	1	0.46	0.46	13.89	17.35	0.0033	0.0005
C (*V*_*ex*_)	0.26	0.26	1	1	0.26	0.26	7.83	9.78	0.0173	0.0055
D (*V*_*Ag*_)	0.19	0.19	1	1	0.19	0.19	5.76	7.20	0.0352	0.0147
E (time)	5.409 ×10^−3^	5.409 ×10^−3^	1	1	5.409 ×10^−3^	5.409 ×10^−3^	0.16	0.20	0.6950	0.6578
AB	4.573 ×10^−3^		1		4.573 ×10^−3^		0.14		0.7183	
AC	0.029		1		0.029		0.86		0.3726	
AD	2. ×10^−4^		1		2.865 ×10^−4^		8.582 ×10^−3^		0.9279	
AE	0.10	0.10	1	1	0.10	0.10	3.11	3.89	0.1055	0.0634
BC	0.086	0.086	1	1	0.086	0.086	2.58	3.22	0.1367	0.0886
BD	0.15	0.15	1	1	0.15	0.15	4.46	5.58	0.0584	0.0291
BE	4.820 ×10^−3^		1		4.820 ×10^−3^		0.14		0.7112	
CD	0.024		1		0.024		0.71		0.4160	
CE	0.18	0.18	1	1	0.18	0.18	5.28	6.60	0.0421	0.0188
DE	0.13	0.13	1	1	0.13	0.13	4.00	5.00	0.0708	0.0376
A^2^	0.092	0.11	1	1	0.092	0.11	2.77	3.97	0.1242	0.0610
B^2^	0.060		1		0.060		1.79		0.2076	
C^2^	0.41	0.44	1	1	0.41	0.44	12.31	16.58	0.0049	0.0007
D^2^	6.989 ×10^−3^		1		6.989 ×10^−3^		0.21		0.6562	
E^2^	9.349 ×10^−3^		1		9.349 ×10^−3^		0.28		0.6072	
**Model**	2.53	2.38	20	12	0.13	0.20	3.78	7.44	0.0137	<0.0001
**Residual**	0.37	0.51	11	19	0.033	0.027				
**Lack of fit**	0.18	0.32	6	14	0.031	0.023	0.84	0.63	0.5891	0.7710
**Pure error**	0.18	0.18	5	5	0.037	0.037				
**Total**	2.89	2.89	31	31						
**Model statistics**										
**R**^**2**^ **(Regression coefficient)**			**R**^**2**^ **(Adjusted)**		**R**^**2**^ **(Predicted)**			**Adequate precision**		
**FM**	**RM**		**FM**	**RM**	**FM**	**RM**		**FM**	**RM**	
0.873	0.825		0.642	0.714	0.783	0.546		10.093	13.823	
**Equations in terms of actual factors**										
**FM**					**RM**					
SPR Intensity =+3.50 ^−^3.97 × 10^−3^ Temp +0.69 [pH] −1.24 [V_ex_] +0.150 [V_Ag_] + 9.90 time –7.51 × 10^−4^ [Temp × pH] +2.83×10^−3^ [Temp × V_ex_] +5.64 × 10^−5^ [Temp × V_Ag_] −8.95 × 10^−5^ [Temp × time] +0.049 [pH × V_ex_] −0.013 [pH × V_Ag_] −1.93 × 10^−4^ [pH × time] −7.73 × 10^−3^ [V_ex_ × V_Ag_] +1.75 × 10^−3^. [V_ex_ × time] −3.045 × 10^−4^ [V_Ag_ × time] +2.50×10^−4^ [Temp^2^] −0.020 [pH^2^] +0.120 [V_ex_^2^] +6.17 × 10^−4^ [V_Ag_^2^] −4.96 × 10^−6^ [time^2^]					SPR Intensity = −1.77 −0.0031 Temp +0.29 [pH] −1.34 [V_ex_] +0.164 [V_Ag_] + 0.0071 [time] −9.00 × 10^−5^ [Temp × time] +0.049 [pH × V_ex_] −0.013 [pH × V_Ag_] +1.75 × 10^−3^ [V_ex_ × time] −3.00 × 10^−4^ [V_Ag_ × time] +2.6510^−4^ [Temp^2^] +0.122 [V_ex_^2^]					

**DF** = Degree of Freedmen, **F** = Fisher-ratio, **R**^**2**^ = Correlation Coefficient, **FM** = Full Model, **RM** = Refined model.

It was shown in Table 3 by ANOVA that the full model (FM) is valid according to the p-value of Fisher F-test to explain the SPR intensity (model responce) properly. In this regard, the low p-value of the model (0.0131) indicated the significance of the mean square of model in comparison to the mean square of residual. Also, the “lack of fit” (LOF) of Eq. (1) is not significant as shown by the p-value of the lack of fit. In spite of these results, it can be observed in Table 3 that the p-values of some of the variables in FM are not significant. According to which is common in CCD modeling, terms with P-values greater than 0.1 are not significant and must be eliminated from the final selected model at the desired confidence limit. Thus a variable selection strategy (backward elimination) was applied to eliminate the redundant variables or interactions in Eq. (1) and development of a refined linear model (RM), as shown in the following:

2 $$\begin{array}{rcl}{\rm{SPR}}\,{\rm{Intensity}} & = & +0.86+0.11\,Temp+0.14\,pH\,-\,0.10\,{V}_{ex}+0.090\,{V}_{Ag}+0.015\,time\\  &  & \,-\,0.081\,Temp\times time+0.073\,pH\times {V}_{ex}\,-\,0.096\,pH\times {V}_{Ag}+0.10\,{V}_{ex}\times time\\  &  & \,-\,0.091\,{V}_{Ag}\times time+0.060\,Tem{p}^{2}+0.12{{V}_{ex}}^{2}\end{array}$$

As it can be seen by comparison of Eqs. (1) and (2), among 20 terms in FM (Eq. 1), five interaction terms such as *Temp × pH, Temp × V*_*ex*_*, Temp × V*_*Ag*_*, pH × time, V*_*ex*_ *× V*_*Ag*_ and three self-interaction variables such as *pH*^2^, *V*_*Ag*_^2^ and *time*^2^ was excluded from full model by backward elimination and Eq. (2) was suggested as the simpler model named as refined model (RM) contained 12 variables (original ones, interactions and self-interactions). However it is very worthy to note that the constraint which was applied during variable selection was obtaining a “hierarchy” model i.e. not removing a variable in presence of each kind of interactions of the removed variable. In other words, the variable “*time*” was retained in model because of presentation of its interactions. Thus As it can be seen in Table 3, the p-value of coefficients of each remaining variables in Eq. (2) as the RM (except “*time*”) are lower than 0.1 which confirms the significance of coefficients. On the other hand the p-value of the total model in RM is also lower than 0.0001 and indicates the significance of obtained linear model as well. Here again, as another good criteria, the p-value of the lack of fit of model suggests that the LOF of Eq. (2) is not significant.

By comparison of FM and RM for the optimization of SPR intensity in Table 3, it can be seen that F-values of the model improved significantly from 3.78 to 7.44. Also F-value of lack of fit in both FM and RM remained below 1 (0.84 and 0.63 respectively) and P-values of LOF increased from 0.5891 to 0.7710 which shows the decreasing of error in reduced model after variable selection. Thus, the Eq. (2) seems acceptable. In addition to the equations obtained based on codded factors shown in Eqs. (1) and (2), the equations obtained from real values of experimental factors (without coding) before and after variable selection are also represented in Table 3.

For more evaluation of the models, the correlation coefficient of the model (R^2^) and adjusted R^2^ (*R*^2^_*adj*_) were calculated which were equal to 0.83 and 0.72 respectively for Eq. (2) which have improvement in compare with Eq. (1) and show enough goodness of fit in Eq. (2)^48^. It is noteworthy that for limited number of runs in models with high number of variables, *R*^2^_*adj*_ is a better parameter than R^2^ because R^2^_adj_ is adjusted to the number of variables and the size of runs (here 32) in model. The *R*^2^_*pred*_ for Eq. (2) was also equal to 0.78 which indicates an acceptable prediction ability^48^. “Adequate precision” is also another statistical indicator for validation of our model and can be an estimate for signal to noise ratio. The “adequate precision” in our suggested refined model (Eq. (2)) was equal to 13.8 which was significantly greater than 4, as the cut-off value for acceptance of precision^49^. Thus, because of noted reasons, the modified model (shown as RM) has been utilized for further process and optimization of AgNPs synthesis. The sings of coefficients in final suggested equation (Eq. 2) for one of the factors (*V*_*ex*_) is negative and for all other four ones (*Temp, pH, V*_*Ag*_ and *time*) are positive (Eq. (2)).

The direct effect of temperature on the SPR may be due to enhancing in the rate of NPs formation^50^. As it was expected increasing the pH has positive effect on the SPR intensity which can be because of increasing the ionization of active functional groups especially phenolic groups in *P. major* extract and enhancing the reduction ability of extract and production of AgNPs. Here, it can be observed that *V*_*Ag*_ have a direct relationship on SPR intensity but but it has been reported in literature that has reverse effect on the size of AgNPs^26,46,51^. Increasing SPR intensity shows enhancing NPs concentration. However it should be emphasized that the proposed model has a multi-parameter nature and the effect of other factors and interactions should be considered simultaneously. The positive sign of time in the proposed model also shows that as the mixing time increased, the formation of AgNPs enhanced as well. The negative sign of V_ex_ indicates that decreasing in volume of *P. major* extract causes increasing in the SPR intensity of the obtained AgNPs. In high volume of *P. major* extract, the SPR intensity decreased and the AgNPs were not stable due to an excess amount of reducing compounds which may cause instantaneous precipitation of particle^52^.

As can be observed in Eq. (2), among the interaction terms, the sign of *Temp × time, pH × V*_*Ag*_ and *V*_*Ag*_ *× time* are negative which shows their reverse effect on AgNPs synthesis and the sign of others (*V*_*ex*_ *× time, Temp*^2^ and *V*_*ex*_^2^) are positive which emplies direct effect on the synthesis quality and subsequently on their SPR intensity.

**Figure 2:**
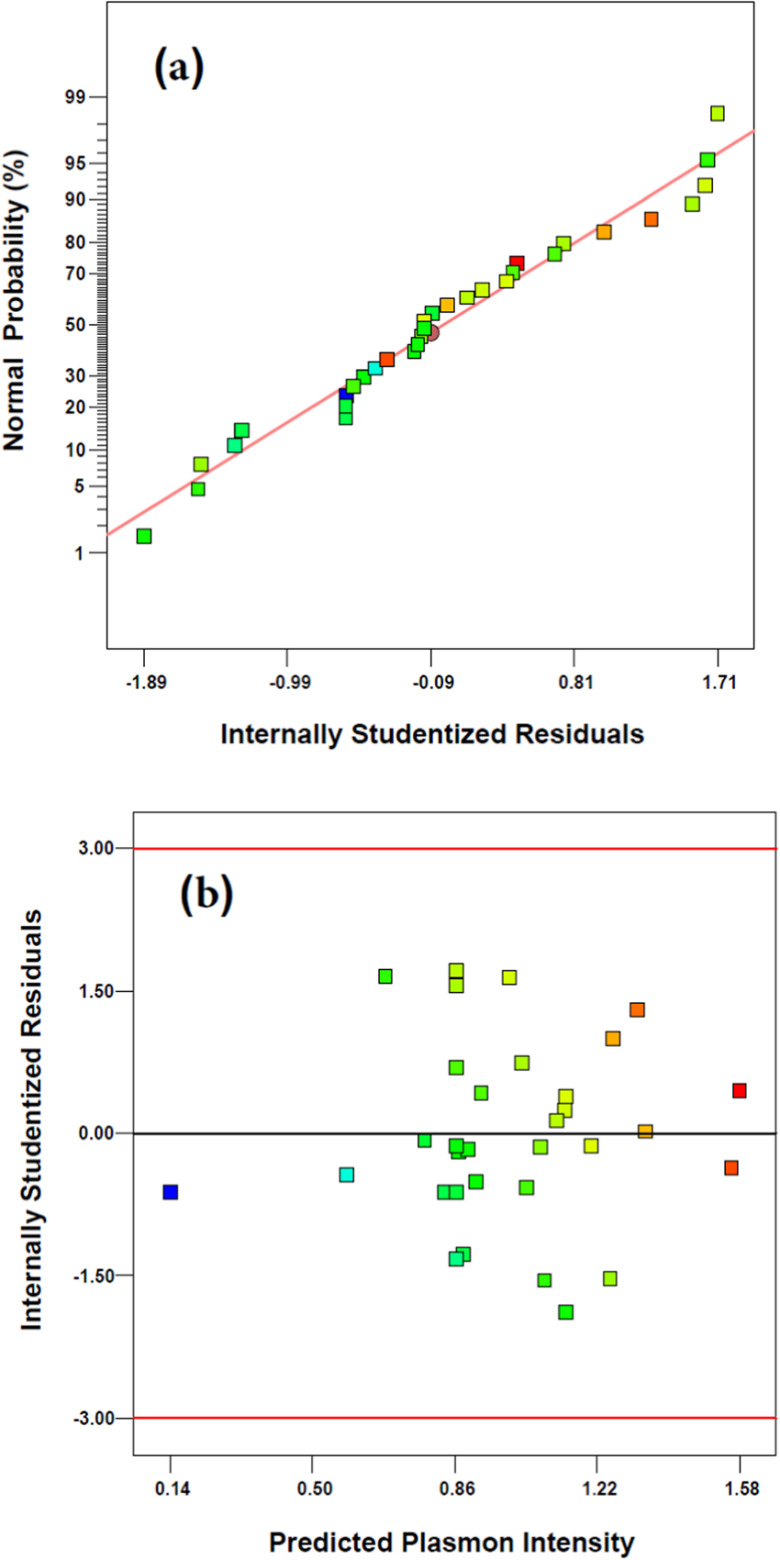
Normal probability plot of internally studentized residuals for the suggested refined quadratic model for SPR intensity (**a**) and plot of studentized residual versus predicted SPR intensity (**b**).

Two additional approaches which are useful to evaluate the accuracy of models are following the normal probability of residual and also the internally studentized residual versus predicted response (SPR intensity). The normal probability plot of the residuals is represented in Fig. 2(a) and shows that all the residual values lie along the straight line with no big deviations and confirms the normality of errors-distribution. It is also shown in Fig. 2(b) that not only the residual are randomly distributed in both sides of zero line (without any systematic pattern in one side), but also lie in the range of ±2σ (even lower than permitted range of ±3σ). Therefore, Fig. 2 declares that the proposed linear model is acceptable and has not any systematic error and can be applied for optimization of the synthesis process.

### Visualization and comparison of interactions

**Figure 3:**
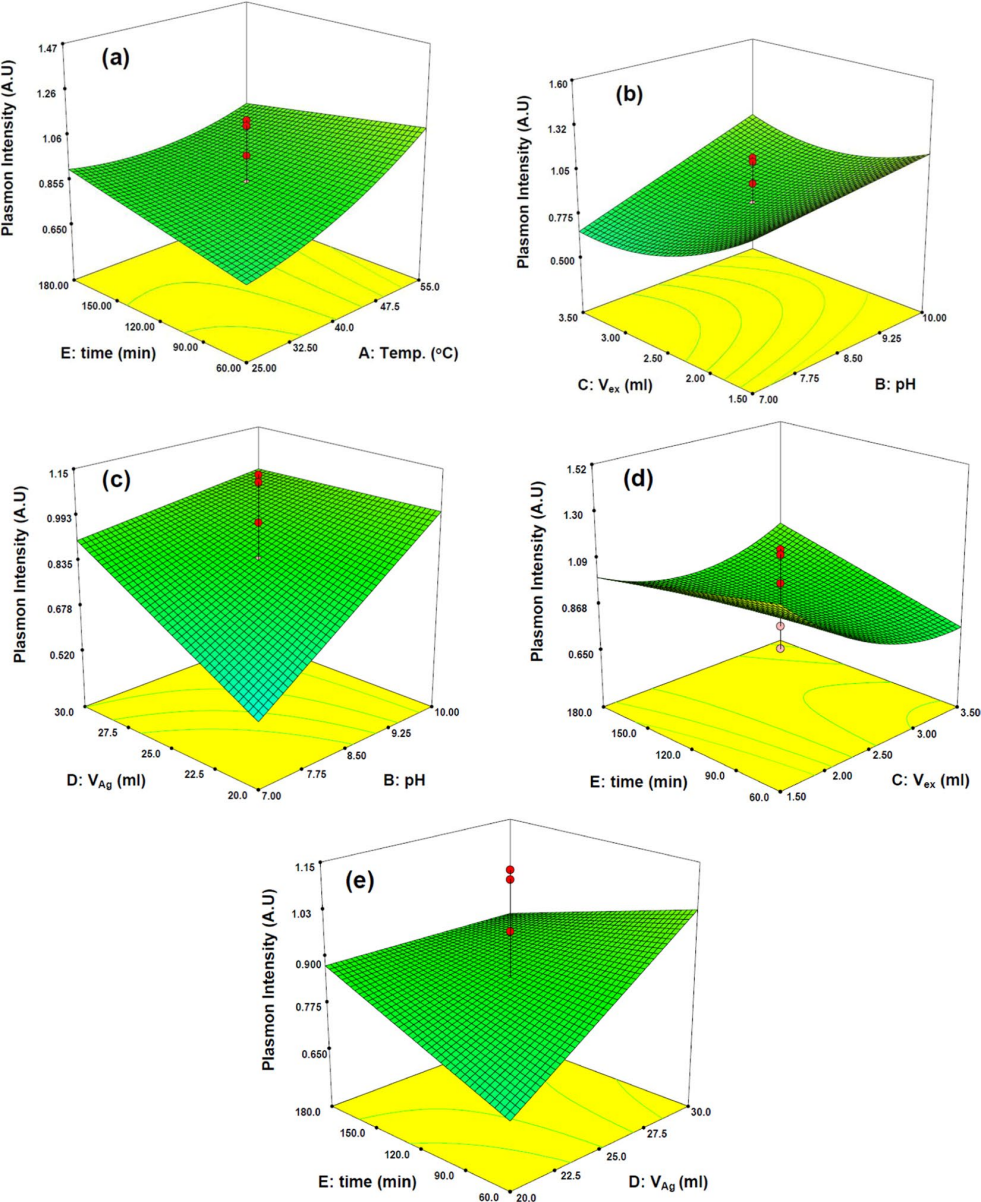
Three dimensional response surface plots of independent factors effect (time–temperature (**a**), volume of extract–*pH* (**b**) volume of Ag solution–*pH* (**c**), *time*–volume of extract (**d**) and *time*–volume of Ag solution (**e**)) on the SPR intensity of AgNPs.

To investigate the effect of various operational factors (independent variables) on SPR intensity of AgNPs (dependent variable), three-dimensional (3D) surface representations of the factors are illustrated in Fig. 3. In this regard, graphs have been prepared by drawing the response surface (i.e. SPR intensity) versus the two effective factors that shows binary interactions whereas other factors have been retained constant at the central level of the applied CCD. Figure 3(a) shows that *Temp* has higher impact on the SPR intensity in compare with *time*, because slope of the obtained surface in the direction of *Temp* axis is steeper than *time* axis. On the other hand, by comparison of Fig. 3(a) with Fig. 3(d,e) it can be observed that the steepest slope of the surface plot can be observed for *time* versus *V*_*Ag*_ and the lowest slope was obtained versus *V*_*ex*_. Interestingly only interaction of *pH* with *V*_*ex*_ and *V*_*Ag*_ were entered in model which are shown in Fig. 3(b,c) respectively. As it was expected and according to Fig. 3(b.c), the synthesis of AgNPs was better done in alkaline pH. It is also clear from Fig. 3(c) that the interaction of *pH* and *V*_*Ag*_ is the biggest effect on the synthesis of AgNPs based on the slope of curve.

According to the Fig. 3(a,c and e), there are no abnormal interaction between factors because all of the obtained surfaces are smooth in each plot and both factors have similar effect on the SPR intensity. It is also clear that, when the factors in Fig. 3(a,c and e) are in their higher possible amount, the SPR intensity and the quality of synthesis increased. But as can be seen in Fig. 3(b,d) the interaction of *pH × V*_*ex*_ and *V*_*ex*_ *× time* are more complex because there is more curvature in their 3D surface. On the other hand the increasing of factors during enhancement of SPR intensity is not observed in Fig. 3(b,d).

### Optimal operational condition of synthesis

**Figure 4:**
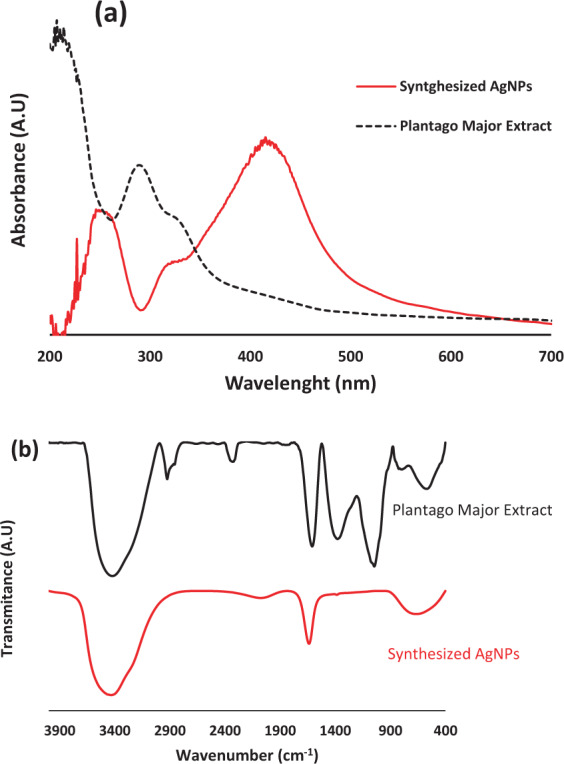
UV-Vis absorption (**a**) and FT-IR transmittance (**b**) spectra of Synthesized AgNPs using *P. Major* seed extract compared with raw extract.

After development and validation of the multivariate linear models, the optimal synthesis conditions have been discovered with a multi-response approach. Here, it was assumed simultaneously to maximize the SPR intensity to have maximum amounts of AgNPs with lower sizes. The predicted optimal conditions was determined to be: *Temp* = 55 °C, *pH* = 9.9,*V*_*ex*_ = 1.5 mL, *V*_*Ag*_ = 30 mL and *time* = 60 min. To check the accuracy of the predicted optimal conditions, some synthesis batches of AgNPs were prepared under the noted condition and one of the obtained UV-Vis spectra from the synthesized particles is demonstrated in Fig. 4(a). Good SPR spectra were obtained from the synthesized NPs. This result illustrates good agreement between predicted values by model and experimental ones for the optimal conditions. For example, in the optimal conditions, the relative errors for prediction of PSR intensity in different synthesis runs were 2–5.5%. These results confirm the ability of *P. major* extract for biosynthesis of AgNPs with good repeatability and also show the possibility of controlling the conditions with a mathematical approach.

### Reduction ability of *Plantago major* extract

In general, the phytochemicals and phenolic compounds in the herbal extracts could be act as the reducing components to convert the Ag^+^ ions into Ag°, to stabilize the synthesized AgNPs and to inhibit agglomeration of NPs via chelate effect base on binding to the metals^53^. The *P. major* extract also acted as the stabilizing agent that obviates the addition of any other chemical reducing agent or external stabilizing compound and surfactant.

The *P. major* extract including alkaloids, flavonoids, phenolic acid derivatives, terpenoids, fatty acids, iridoid glycosides, polysaccharides and vitamins^54^. The main flavonoids present are flavones, including apigenin and luteolin^55,56^. Plantamajoside and acteoside, also named as verbacoside are the determined caffeic acid derivatives in *P. major* extract^54^. Thus, the utilized plant extract is a good option for reduction of silver ion and formation of AgNPs.

To have an estimate on the functional groups of the *P. major* extract FT-IR and UV-Vis spectroscopy were carried out and these spectra are shown in Fig. 4. The UV-Vis spectrum of *P. major* extract in aqueous media is shown in Fig. 4(a). The bonds ranging 262 to 356 nm with λ_max_ at 315 nm can be related to the transition within the cinnamoyl ring system and the double bonds (i.e. π → π* transitions)^57^. It is noteworthy that the extract of *P. major* was prepared in aqueous media and thus the highly polar phytochemical compounds such as polyphenolics and some alkaloids are extracted from seeds via water thus it is contained many conjugated aromatic species and double bonds. Therefore, the bond which is appeared with a maximum around 206 nm is originated from the benzoyl system and π → π* transitions due to the existence of polyphenolics^57–59^.

FT-IR spectroscopy for the extract was also carried out to appear the possible bio-compounds with reducing ability to reduce the Ag^+^ ions and to stabilize the constructed AgNPs by utilized herbal extract. As can be observed in Fig. 4(b), there are some transmission peaks at 3440, 2926, 2338, 1600, 1360, 1050, 790, 588 cm^−1^. The band in 3600 to 3400 cm^−1^ is an indicator of stretching in OH group within free hydroxyl or involved in hydrogen bonds and the broad bonds z in range of 3500 to 3200 cm^−1^ is also an indicator of Ar–OH or –OH group bond in aromatic structures which confirm the existence of phenolic compounds in *P. major* extract. On the other hand the band in 3440 cm^−1^ can be a representative of stretching in N-H group. The band in 2926 cm^−1^ is appeared in the indicator range of carboxylic acids. The band in 1600 cm^−1^ is another representative range of carboxylic acids (1610 to 1550 cm^−1^) and the band in 1600 cm^−1^ can be also a typical band of conjugated dienes. The band in 1360 cm^−1^ can show the S=O band or N–O group. The observed bond in 1050 cm^−1^ shows the alcohols in the utilized herbal extract and the band in 790 cm^−1^ illustrates “out-of-plane stretching” in C=C or out-of-plane stretching of C–H in aromatics or S–OR within ester compounds. The band in 588 cm^−1^ are appeared around 600 cm^−1^ which representative of bending in C=C bond of alkenes or stretching in alkyl halides. Based on results, the flavonoid and other phenolic molecules in the *P. major* extract can act as reducing agent of Ag^+^ ions and stabilization of the constructed NPs.

### Stability and reproducibility of nanoparticles

The stability of the AgNPs (synthesized in optimum conditions) was checked by storing the colloidal dispersion of NPs in dark and at 4 °C and measuring its absorption spectra during time. After 30 days, the dispersion was stable and no considerable spectral changes and precipitation were detected which showed its stability in the investigated time period (data not shown). The synthesis of NPs was repeated more than 10 times in optimum conditions and the characteristics were reproducible.

### Characterization of AgNPs

#### XRD analysis

**Figure 5:**
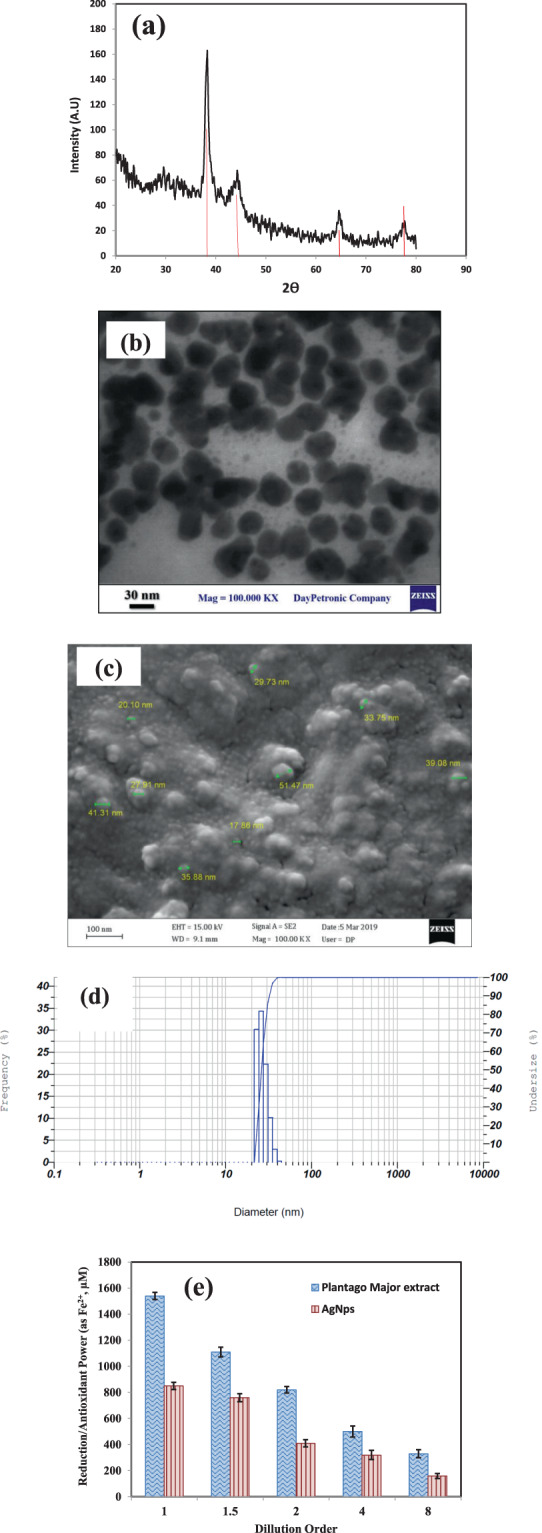
XRD patterns (**a**), TEM image (**b**), SEM micrographs (**c**), DLS histogram (**d**) of the biosynthesized AgNPs(**d**), and FRAP assay results to evaluate antioxidant potential of the *P. major* seed extract and synthesized AgNPs (**e**).

The XRD of biosynthesized AgNPs was recorded at 2θ values ranging from 20°–80° to clarify the crystal structure of NPs which is represented in Fig. 5(a). XRD analysis showed distinct peaks at 2θ equal to 38.1°, 44.2° and 64.5° and 77.4°, which indicated the planes (1 1 1), (2 0 0), (2 2 0) and (3 1 1) of the faced center cubic (fcc) silver respectively which determines the crystalline nature of the prepared AgNPs using *P. major* extract^60^. Thus, the obtained diffraction pattern showed that the most abundant crystals of the prepared NPs are in (111) plane. The average crystalline size was also calculated based on Scherrer’s equation which was equal to 11 nm^61^.

#### TEM and SEM

The size and morphology of the prepared AgNPs was investigated utilizing TEM image which can be observed in Fig. 5(b). TEM images show relatively uniform particles in diameter with almost spherical shape and well-distributed particles. An estimation was also done on the particle size of NPs based on different recorded images and the range of size was found between 10 nm to 39 nm with an average size of 23.5 nm. A SEM micrograph of the dry mass of synthesized AgNPs is shown in Fig. 5(c), which represents NPs with an average size of ~30 nm.

#### Dynamic Light Scattering

The size distribution of the obtained AgNPs in the optimum conditions was also checked with DLS as a standard particle size analyzer. As it is represented in Fig. 5(d), 10.0% of AgNPs was of the sized of 22.4 nm, 50% of the size of 26.1 nm and 90% of the size of 32.3 nm which results in agreement with the SEM and TEM studies.

#### FTIR spectra of NPs

FTIR has been used as an important method to find the responsible functional groups in stabilizing and surface modifying of NPs^62^. In addition to the FTIR spectrum of *P. major* extract which was discussed in previous parts, the FTIR spectrum of biosynthesized AgNPs was recorded which is represented in Fig. 4(b). The FT-IR of AgNPs indicates three representative peaks at 3475, 1652 and 714 which has some changes in shape and position in compare with to the observed peaks in the *P. major* extract. This can be due to the interaction between silver(I) ions and active sites of phytochemical compounds during stabilizing and producing of AgNPs. Appearance and shift in the position of peaks at 3600–3200 cm^−1^, 1610–1550 cm^−1^ and 714 cm^−1^, indicated the presence of OH functional groups, carboxylic acid, C=C aliphatic and probably amine groups in the capping agents which act as the stabilizers of the synthesized AgNPs using *P. major*. The main possible adsorbed compounds on the AgNPs surface can be polyphenolic molecules probably by π-electrons interaction in the absence of common strong ligating agents. These results confirmed the reduction of Ag^+^ ions to Ag° and capping the surface of produced NPs by the natural polyphenolic materials in *P. major* extract^61^

### Ferric-Reducing/Antioxidant Power

Because of bioactive compounds in the *P. Major*, the reducing ability of the *P. major* extract and biosynthesized AgNPs capped by extract was checked and the results are illustrated in Fig. 5(e). It is observed from this figure that, the reducing activity enhanced by increasing the amount of *P. major* extract or the produced biosynthesized AgNPs and a dose-dependent manner was observed in this study. The similar result also reported by other researchers and also by our research group in synthesized AgNPs using other herbal extracts^26,63^. The phytochemical constituent in the *P. major* extract, which is effective in the bioreduction of Ag^+^ to Ag^0^, can be appeared by the FRAP assay. The FRAP values were obtained in range of 303 to 1540 mM (as Fe (II)) dependent on different diluted sample of *P. major* extract (concentrations=134.75 –1078 μg/ml), which can be due to the high concentration of phenolic compounds and flavonoids in the extract. This can be also an evidence to show that the compounds in *P. major* extract were responsible in the reduction of Ag^+^ and construction of AgNPs^64^. As it was observed in results, the synthesized AgNPs has also acceptable reducing ability which can be related to the polyphenolic compounds in the *P. major* extract encapsulated on the surface of NPs^65^.

CCD was done for optimizing the effective parameters in the synthesis of AgNPs. The hypothesis was obtaining highest quality of synthesis based on monitoring the SPR of the NPs and the response vector of CCD was not any one of biological activities. But, to evaluate the relationship between the SPR and anti-oxidant activity in FRAP assay, the FRAP assay was performed in some runs in Table 2 (i.e runs #4, #5, #15, #16, #18 and #20), with various SPR intensity from low to high. It was observed that the antioxidant power in the optimum run was bigger than all of these studied runs (data not shown). On the other hand among these six runs, run #20 had higher activity and run#5 has the lowest value which denote the effect of SPR on the anti-oxidant power in FRAP assay.

### Antimicrobial activity

**Figure 6:**
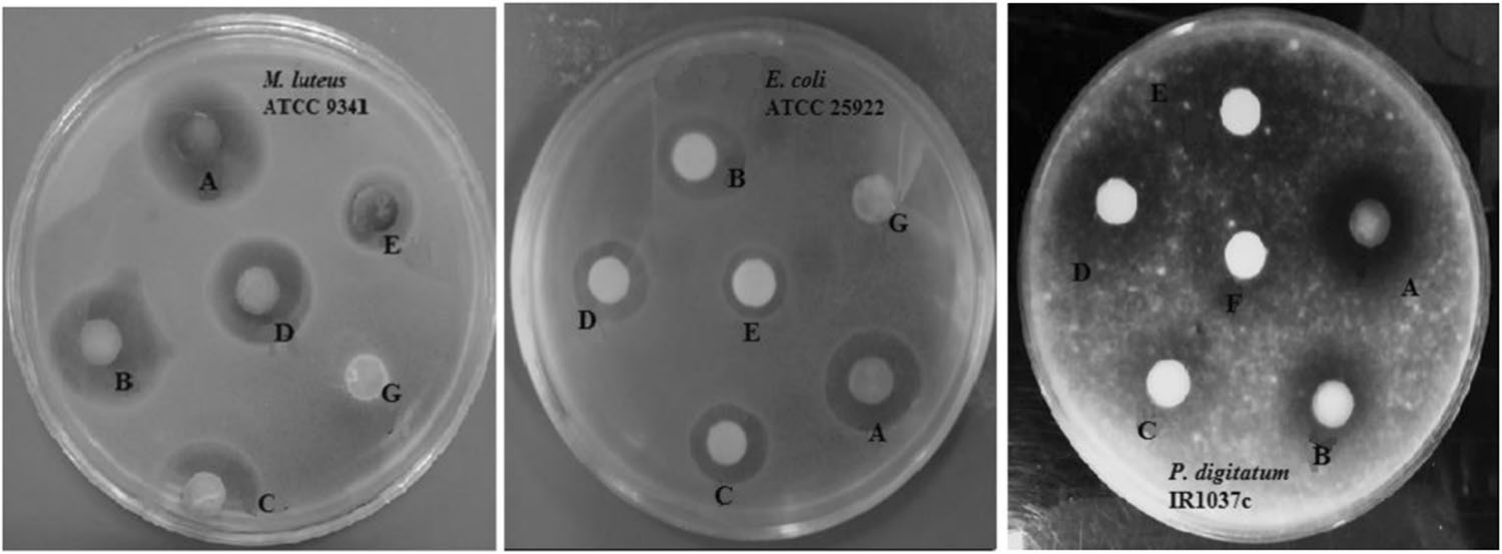
Antimicrobial activity of AgNPs against *Micrococcus luteus, Escherichia coli*, and *Penicillium digitatum* at different concentrations: (**A**) full strength (100%), (**B**) 50%, (**C**) 25%, (**D**) 12.5%, (**E**) 6.2%, and (**F**) 3.1% (v/v) [Note: (**G**) *Plantago major* seed extract].

The antibacterial effect of different strengths of AgNPs evaluated by disk diffusion method is represented in Table 4. Compared with *E. coli*, *M. luteus* was sensitive to lower concentrations of AgNPs which was confirmed by the MIC levels. The concentrations of 12.5% and 25% (v/v) were recorded as the MIC for *M. luteus* and *E. coli*, respectively. Although several studies have shown a remarkable activity of nano-silver against *E. coli* compared to the Gram-positive strain *Staphylococcus aureus*^66–68^, its effect is highly dependent on the species/strain variation in uptake, general susceptibility and tolerance mechanism^42^. In the study conducted by Kumar *et al*.^42^, *M. luteus* and *Bacillus subtilis* were found to be the most sensitive strains to biosynthesized AgNPs using *Couroupita guianensis* flower buds extract, while *S. aureus* exhibited much more resistance to AgNPs than *E. coli*. As shown in Fig. 6, AgNPs also revealed antagonistic activity against the indicator mold *P. digitatum* at all the concentrations tested with inhibition zone diameters of 15.5–16.5 mm. The aqueous extract of *P. major* was ineffective on all test organisms. The inefficacy of aqueous extract of *P. major* against *E. coli* has been reported by other researchers as well^69,70^. Therefore, the inhibitory effects found in this study can most likely be attributed to AgNPs. There are several reports on the antimicrobial activity of AgNPs synthesized using plant extracts against pathogenic bacteria and fungi^66–68,71–73^. The antimicrobial effects of AgNPs can be at least partly due to their ability to bind to bacterial cell membrane which may in turn lead to the increased membrane permeability^66^. Moreover, they may disrupt enzymatic activity of bacteria by interacting with the sulfhydryl group (-SH) of bacterial enzymes^71^.

**Table 4:** Antimicrobial activity of AgNPs synthesized by *Plantago major* seed extract determined by disk diffusion method.

AgNPs concentration/strength (%)	Mean inhibition zone diameter* (mm)	
	Micrococcus luteus ATCC 9341	Escherichia coli ATCC 25922
100.0	17.1	15.1
50.0	16.1	13.9
25.0	15.6	12.9
12.5	14.5	12.2
6.2	11.5	10
3.1	10	0
1.6	0	0

*Values are given as mean of three replicates.

To evaluate the relationship between the SPR and antibacterial activity, the activity against *M. luteus* were evaluated for some of runs in Table 2, having various SPR intensities (form low to high). It was observed that higher SPR can cause higher inhibition zone. For example the inhibition zone for run#4 (I.Z. = 10 mm), run#5 (I.Z. = 0 mm), run#15 (I.Z. = 0 mm), run#16 (I.Z. = 9 mm), run # mm), run #20 (I.Z. = 14 mm) confirmed this point. It was also observed that the antibacterial activity in the optimum run was bigger than all of these studied runs (see Table 4 for comparison).

## Conclusion

CCD as a known RSM can be used to optimize the experimental conditions of the biosynthesis process of AgNPs using herbal extracts. In this approach the interaction of factors and also the original factors (temperature, pH, volume of extract used for synthesis, volume of AgNO_3_ solution and synthesis time) can be covered during NPs preparation/optimization simultaneously and the intensity of SPR spectra of the AgNPs was used as the response (dependent variable). A linear model with R^2^ = 0.825 with statistical significance in ANOVA as well as not-significant lack of fit showed the efficiency of CCD in biosynthesis of AgNPs. In optimum conditions, (*Temp* = 55 °C, *pH* = 9.9, volume of *P. major* extract=1.5 mL, volume of silver salt=30 mL, time of synthesis= 60 min), stable and high quality AgNPs were prepared. It is worthy to note that involving the interaction between experimental factors in the obtained model indicates the importance of multi-parameter optimization and its potential in compare with one-at-a-time optimization in nano synthesis systems as complex media.

The biosynthesis of AgNPs using extract of *P. major* seeds can be an effective method in pH ~10. FTIR showed that the main possible adsorbed compounds on the AgNPs’ surface can be polyphenolic moleculesprobably by π-electrons interaction without common strong ligating agents. Because of bioactive compounds in the *P. major*, the reducing power of the *P. major* extract and biosynthesized AgNPs was evaluated using FRAP assay. The FRAP values indicate a dose dependent response and the reducing activity was increased in higher concentration of *P. major* seed extract or final AgNPs which shows its possible anti-oxidant ability.

DLS confirmed that 10.0% of the prepared AgNPs using this method was of the sized of 22.4 nm, 50% of the size of 26.1 nm and 90% of the size of 32.3 nm, which was in agreement with the SEM and TEM studies. XRD also confirmed the potential of the *P. major* seeds extract in the reduction of Ag^+^ and preparation of Ag° NPs with average crystalline size around ~11 nm.

According to the best of our knowledge this is also the first work on the evaluation of antibacterial and antifungal activity of AgNPs synthesized by *P. major* seeds extract which showed an acceptable activity against *Micrococcus luteus*, *Escherichia coli*, and *Penicillium digitatum*. However the antibacterial activity was not tested against other kinds of bacteria and fungi which is a limitation of this work and should be done in future studies.

Since green synthesis of AgNPs is cost-effective, environmental friendly and compatible with human health due to less waste and safer products, this method can be considered as an alternative method of synthesis. Because of safe process of synthesis and the confirmed and antioxidant, antibacterial and antifungal activities of AgNPs synthesized by *Plantago* seeds,, the product can be used in medical applications such as surgical gowns, face masks and arms/ knees braces in the future studies. However various papers have reported the antibacterial, antifungal and antioxidant activity of the AgNPs, but the difference of the herbal extract which is used in the current work is popularity and availability of *Plantago major* in society and for people who are familiar with traditional herbals. It can make it a power point for general applications in society especially as antimicrobial agents. Thus, not only the elimination of chemical agents with toxic properties but also, covering both reducing and stabilizing agents by *Plantago major*, as a popular plant with known biological activity, can introduce it as a reliable method. On the other hand the production of AgNPs using the suggested method does not need high temperature and can be completed within one hour.
